# Supplementary material for: Native proline-rich motifs exploit sequence context to target actin-remodeling Ena/VASP protein ENAH
Source: eLife. 2022 Jan 25;11:e70680. doi: 10.7554/eLife.70680 (PMC8789275; doi:10.7554/eLife.70680)
Supplement: Supplementary file 2. [file elife-70680-supp2.docx]

**Supplementary File 2. Dissociation constants for MassTitr peptides derived from human proteins binding to monomeric ENAH EVH1 domain**. In light blue and orange are peptides annotated as “high affinity” or “low affinity” by MassTitr. A peptide from ActA (gray) was used as a positive control.

| **Name** | **Sequence** | **K_D_ (μM)** ^a^ |
| --- | --- | --- |
| PCARE | AAKSEELSCEMEGNLEH**LPPPP**MEVLMDKSFASLES | 0.18$\pm$ 0.04 |
| ABI1 | FDD**FPPPP**PPPPVDYEDEEAAVVQYNDPYADGDPAW | 2.6 $\pm$ 0.6 |
| LPP | KQPGGEGDF**LPPPP**PPLDDSSALPSISGN**FPPPP**PL | 4.7 $\pm$ 2.4 |
| ZYX | ALGGA**FPPPP**PPIEES**FPPAP**LEEEI**FPSPP**PPPEE | 5.0 $\pm$ 1.0 |
| ACTA | GFNAPATSEPSSFE**FPPPP**TEDELEIIRETASSLDS | 4.9$\pm$ 0.5 |
| NHSL1 | ADRSPF**LPPPP**PVTDCSQGSPLPHSPV**FPPPP**PEAL | 9.7$\pm$ 2.5 |
| SHROOM3 | VYSMDD**FPPPP**PHTVCEAQLDSEDPEGPRPSFNKLS | 9.3 $\pm$ 0.02 |
| ABI3 | SPPPPDEELPLPLD**LPPPP**PLDGDELG**LPPPP**PGFG | 13.6 $\pm$ 1.9 |
| KIAA1522 | PPAPEEQDLSMADFPPPEEAFFSVASPEPAGPSGSP | 13.6 $\pm$ 0.7 |
| FBLIM1 | PPVLDGEDVLPDLDL**LPPPP**PPPPVLLPSEEEAPAP | 16.0 $\pm$ 0.3 |
| TNK2 | TPVVDWDARP**LPPPP**AYDDVAQDEDDFEICSINSTL | 21.3$\pm$ 0.2 |
| TJAP1 | PEEELPLPAFEKLNPYPTPSPPHPLYPGRRVIEFSE | 23.0 $\pm$ 0.2 |
| FYB1 | SGSGGI**FPPPP**DDDIYDGIEEEDADDG**FPAPP**KQLD | 24.3 $\pm$ 0.7 |
| NUTM2E | VVPVMAAQVVGGTQACEGGWSQG**LPLPP**PPPPAAQL | 33.9$\pm$ 1.2 |
| FHOD1 | SVPPPPP**LPPPP**PIKGP**FPPPP**PLPLAAPLPHSVPD | 50.4$\pm$ 8.9 |
| SYNPO2 | KSPIAD**FPAPP**PYSAVTPPPDAFSRGVSSPIAGPAQ | 52.1$\pm$ 4.9 |
| TENM1 | GSTQDVQSSPHNQFTFRP**LPPPP**PPPHACTCARKPP | 62.9$\pm$ 12.5 |

^a^ Affinities determined by BLI as described in the methods. Errors are the standard deviation of 2-3 replicates (see source data file).
