# Supplementary material for: Native proline-rich motifs exploit sequence context to target actin-remodeling Ena/VASP protein ENAH
Source: eLife. 2022 Jan 25;11:e70680. doi: 10.7554/eLife.70680 (PMC8789275; doi:10.7554/eLife.70680)
Supplement: Supplementary file 3. [file elife-70680-supp3.docx]

**Supplementary File 3. Dissociation constants for ABI1-derived peptides binding to ENAH EVH1 domain.** Errors reported as the standard deviation of two or three replicates.

| **Name** | **Sequence** | **K_D_ (μM)**^a^ |
| --- | --- | --- |
| ABI1 | FDD**FPPPP**PPPPVDYEDEEAAVVQYNDPYADGDPAW | 2.4 $\pm$ 0.1 |
| ABI1 b | FDD**FPPPP**PPPPVDYEDEEAAV | 3.9 $\pm$0.9 |
| ABI1 a | FDD**FPPPP**PPPPVDYED | 4.3 $\pm\text{0.2}$ |
| ABI1 ala | FDD**FPPPP**PPPPVAYAA | 8.5 $\pm$ 0.9 |
| FDDFP_8_ | FDD**FPPPP**PPPP | 9.5 $\pm$ 0.9 |
| FP_8_ | **FPPPP**PPPP | 28.4 $\pm$ 5.7 |
| FP_4_S_4_ | **FPPPP**SSSS | 110 $\pm$ 9.2 |

^a^ Affinities determined by BLI as described in the methods.
