# Supplementary material for: Native proline-rich motifs exploit sequence context to target actin-remodeling Ena/VASP protein ENAH
Source: eLife. 2022 Jan 25;11:e70680. doi: 10.7554/eLife.70680 (PMC8789275; doi:10.7554/eLife.70680)
Supplement: Supplementary file 4. [file elife-70680-supp4.docx]

**Supplementary File 4. Dissociation constants for peptides derived from human proteins binding to monomeric EVH1 domains from ENAH, VASP, and EVL**.^a^

| **Name** | **Sequence** | **ENAH K_D_ (μM)** | **VASP K_D_ (μM)** | **EVL K_D_ (μM)** |
| --- | --- | --- | --- | --- |
| ActA | GFNAPATSEPSSFE**FPPPP**TEDELEIIRETASSLDS | 4.9$\pm$ 0.5 | 10.9 $\pm$ 0.5 | 2.8 $\pm$ 0.3 |
| ABI1 | FDD**FPPPP**PPPPVDYEDEEAAVVQYNDPYADGDPAW | 2.6 $\pm$ 0.6 | 14.1 $\pm$1.0 | 12.3 $\pm\text{0.}$3 |
| LPP | KQPGGEGDF**LPPPP**PPLDDSSALPSISGN**FPPPP**PL | 4.7 $\pm$ 2.4 | 14.4 $\pm$0.4 | 31.6 $\pm\text{2}\text{.4}$ |

^a^ Affinities obtained using BLI as described in the methods. Errors are the standard deviation of three replicates.
