# Supplementary material for: Native proline-rich motifs exploit sequence context to target actin-remodeling Ena/VASP protein ENAH
Source: eLife. 2022 Jan 25;11:e70680. doi: 10.7554/eLife.70680 (PMC8789275; doi:10.7554/eLife.70680)
Supplement: Supplementary file 5. [file elife-70680-supp5.docx]

**Supplementary File 5. Comparison of affinities of single- and dual-motif peptides for monomeric ENAH WT vs. ENAH R47A.**^a^

| **Name** | **Sequence** | **WT K_D_ (μM)** | **R47A K_D_ (μM)** |
| --- | --- | --- | --- |
| PCARE | AAKSEELSCEMEGNLEH**LPPPP**MEVLMDKSFASLES | 0.18$\pm$ .04 | 0.32 $\pm$ .04 |
| LPP^**^ | KQPGGEGDF**LPPPP**PPLDDSSALPSISGN**FPPPP**PL | 4.7$\pm$ 2.4 | 60.1$\pm$ 6.7 |
| ZYX^**^ | ALGGA**FPPPP**PPIEES**FPPAP**LEEEI**FPSPP**PPPEE | 5.0 $\pm$ 1.0 | 27.3 $\pm$ 2.6 |
| ActA | GFNAPATSEPSSFE**FPPPP**TEDELEIIRETASSLDS | 4.9 $\pm$ 0.5 | 6.6 $\pm$ 2.4 |
| NHSL1^**^ | ADRSPF**LPPPP**PVTDCSQGSPLPHSPV**FPPPP**PEAL | 9.7$\pm$ 2.5 | 51.5 $\pm$ 10.0 |

^a^ Affinities determined by BLI as described in the methods. Errors reported as the standard deviation of three replicates.

^**^ Statistically significant differences in affinity between WT and R47A with p < 0.01.
