# Supplementary material for: Native proline-rich motifs exploit sequence context to target actin-remodeling Ena/VASP protein ENAH
Source: eLife. 2022 Jan 25;11:e70680. doi: 10.7554/eLife.70680 (PMC8789275; doi:10.7554/eLife.70680)
Supplement: Supplementary file 6. [file elife-70680-supp6.docx]

**Protein Constructs for *Native proline-rich motifs exploit sequence context to target actin-remodeling Ena/VASP protein ENAH***

**pDW363 constructs**

Constructs used for bacterial display

**ENAH tetramer**

MSEQSICQARAAVMVYDDANKKWVPAGGSTGFSRVHIYHHTGNNTFRVVGRKIQDHQVVINCAIPKGLKYNQATQTFHQWRDARQVYGLNFGSKEDANVFASAMMHALEVLNSQEAGGGGGGGSACEGLDYDRLKQDILDEMRKELAKLKEELIDAIRQELSKSNTAGSGSGSGLNDIFEAQKIEWHEDTGGSSHHHHHHG*

**Cysteine-less ENAH tetramer**

MSEQSIAQARAAVMVYDDANKKWVPAGGSTGFSRVHIYHHTGNNTFRVVGRKIQDHQVVINSAIPKGLKYNQATQTFHQWRDARQVYGLNFGSKEDANVFASAMMHALEVLNSQEAGGGGGGGSASEGLDYDRLKQDILDEMRKELAKLKEELIDAIRQELSKSNTAGSGSGSGLNDIFEAQKIEWHEDTGGSSHHHHHHG*

SUMO-peptide fusions for ITC and BLI

**SUMO only (empty)**

MAGGLNDIFEAQKIEWHEDTGGSSHHHHHHGSGSGSDSEVNQEAKPEVKPEVKPETHINLKVSDGSSEIFFKIKKTTPLRRLMEAFAKRQGKEMDSLTFLYDGIEIQADQTPEDLDMEDNDIIEAHREQIGG*

**SUMO-ActA**

MAGGLNDIFEAQKIEWHEDTGGSSHHHHHHGSGSGSDSEVNQEAKPEVKPEVKPETHINLKVSDGSSEIFFKIKKTTPLRRLMEAFAKRQGKEMDSLTFLYDGIEIQADQTPEDLDMEDNDIIEAHREQIGGGFNAPATSEPSSFEFPPPPTEDELEIIRETASSLDS*

**All other peptides used in the experiment were cloned into the C-terminus of the following sequence:**

MAGGLNDIFEAQKIEWHEDTGGSSHHHHHHGSGSGSDSEVNQEAKPEVKPEVKPETHINLKVSDGSSEIFFKIKKTTPLRRLMEAFAKRQGKEMDSLTFLYDGIEIQADQTPEDLDMEDNDIIEAHREQIGGSGSG[PEPTIDE SEQUENCE]

Peptide sequences inserted after the C-terminus are listed in Tables 1, S2, and S3

**pMCSG7 constructs**

Constructs used for BLI

**ENAH EVH1**

MHHHHHHSSGVDLGTENLYFQSNAMSEQSICQARAAVMVYDDANKKWVPAGGSTGFSRVHIYHHTGNNTFRVVGRKIQDHQVVINCAIPKGLKYNQATQTFHQWRDARQVYGLNFGSKEDANVFASAMMHALEVL*

**VASP EVH1**

MHHHHHHSSGVDLGTENLYFQSNAMSETVICSSRATVMLYDDGNKRWLPAGTGPQAFSRVQIYHNPTANSFRVVGRKMQPDQQVVINCAIVRGVKYNQATPNFHQWRDARQVWGLNFGSKEDAAQFAAGMASALEALE*

**EVL EVH1**

MHHHHHHSSGVDLGTENLYFQSNAMSEQSICQARASVMVYDDTSKKWVPIKPGQQGFSRINIYHNTASSTFRVVGVKLQDQQVVINYSIVKGLKYNQATPTFHQWRDARQVYGLNFASKEEATTFSNAMLFALNIMNSQE*

Constructs used for crystallography

**ENAH EVH1-ABI1**

MHHHHHHSSGVDLGTENLYFQSNAMSEQSICQARAAVMVYDDANKKWVPAGGSTGFSRVHIYHHTGNNTFRVVGRKIQDHQVVINCAIPKGLKYNQATQTFHQWRDARQVYGLNFGSKEDANVFASAMMHALEVLGGSGSGFDDFPPPPPPPPVDYEDEEAAVVQYNDPYADGDPAW*

**eCPX constructs**

**All peptide sequences, and the T7-pep library, were inserted into the following background:**

MKKIACLSALAAVLAFTAGTSVAGGQSGQSGDYNKNQYYGITAGPAYRINDWASIYGVVGVGYGKFQTTEYPTYKHDTSDYGFSYGAGLQFNPMENVALDFSYEQSRIRSVDVGTWILSVGYRFGSKSRRATSTVTGGYAQSDAQGQMNKMGGFNLKYRYEEDNSPLGVIGSFTYTEKSRTASGGGSGGGSDYKDDDDKGGGSGGGSG**IPLR**[PEPTIDE SEQUENCE]**RIAR**GSGSEQKLISEEDL*

Bold sequences IPLR and RIAR constitute part of the EcoRI and XhoI restriction enzyme sites, respectively.

Peptide sequences are as follows:

| **Name** | **Sequence** |
| --- | --- |
| Empty | No peptide inserted |
| ActA | GFNAPATSEPSSFEFPPPPTEDELEIIRETASSLDS |
| Vinculin | EAFQPQEPDFPPPPPDLEQLRLTDELAPPKPPLPEG |
| SHIP2 | VGEGSSSDEESGGTLPPPDFPPPPLPDSAIFLPPSL |
| OLIG3 | HWAGLPCPCTICQMPPPPHLSALSTANMARLSAESK |
| TRIM1 | ILSGLPAPDFIDYPERQECNCRPQESPYVSGMKTCH* |
