# Supplementary material for: Native proline-rich motifs exploit sequence context to target actin-remodeling Ena/VASP protein ENAH
Source: eLife. 2022 Jan 25;11:e70680. doi: 10.7554/eLife.70680 (PMC8789275; doi:10.7554/eLife.70680)
Supplement: Supplementary file 8. [file elife-70680-supp8.docx]

**Supplementary File 8. Refinement table for ENAH-ABI1 structure**

|  | **ENAH-ABI1 (7LXE)** |
| --- | --- |
| **Wavelength** |  |
| **Resolution range** | 38.74 - 1.88 (1.947 - 1.88) |
| **Space group** | C 2 2 21 |
| **Unit cell** | 56.067 77.485 66.167 90 90 90 |
| **Total reflections** | 157527 (15746) |
| **Unique reflections** | 12056 (1185) |
| **Multiplicity** | 13.1 (13.1) |
| **Completeness (%)** | 98.91 (98.08) |
| **Mean I/sigma(I)** | 15.74 (0.92) |
| **Wilson B-factor** | 47.69 |
| **R-merge** | 0.06278 (2.462) |
| **R-meas** | 0.0654 (2.561) |
| **R-pim** | 0.01806 (0.6999) |
| **CC1/2** | 0.999 (0.743) |
| **CC*** | 1 (0.923) |
| **Reflections used in refinement** | 11941 (1176) |
| **Reflections used for R-free** | 1196 (119) |
| **R-work** | 0.2447 (0.5842) |
| **R-free** | 0.2761 (0.7129) |
| **CC(work)** | 0.954 (0.735) |
| **CC(free)** | 0.964 (0.573) |
| **Number of non-hydrogen atoms** | 942 |
| **macromolecules** | 942 |
| **solvent** |  |
| **Protein residues** | 122 |
| **RMS(bonds)** | 0.006 |
| **RMS(angles)** | 0.76 |
| **Ramachandran favored (%)** | 96.61 |
| **Ramachandran allowed (%)** | 3.39 |
| **Ramachandran outliers (%)** | 0.00 |
| **Rotamer outliers (%)** | 0.00 |
| **Clashscore** | 2.17 |
| **Average B-factor** | 75.74 |
| **macromolecules** | 75.74 |
| **solvent** |  |

Statistics for the highest-resolution shell are shown in parentheses.

Note that the ABI1 FP_8_ peptide is numbered 120-129 in accordance with its respective fusion protein numbering.
